# Plants used by the rural community of Bananal, Mato Grosso, Brazil: Aspects of popular knowledge

**DOI:** 10.1371/journal.pone.0210488

**Published:** 2019-01-30

**Authors:** Graciela da Silva Miguéis, Rosa Helena da Silva, Geraldo Alves Damasceno Júnior, Germano Guarim-Neto

**Affiliations:** 1 Instituto de Ciências Exatas e Naturais, Universidade Federal de Mato Grosso, Rondonópolis, Mato Grosso, Brasil; 2 Instituto de Biociências, Universidade Federal de Mato Grosso do Sul, Campo Grande, Mato Grosso do Sul, Brasil; 3 Instituto de Biociências, Universidade Federal de Mato Grosso, Cuiabá, Mato Grosso, Brasil; College of Agricultural Sciences, UNITED STATES

## Abstract

Studies in rural communities are important to maintain popular knowledge between generations, as well as to identify new species for pharmaceutical production. Thus, the objectives of this study were to determine which plant species the rural community of Bananal, Mato Grosso, Brazil, uses by calculating the levels of fidelity and concordance regarding species uses among residents and to determine if there is a relationship between the number of known useful plants and levels of education, age, and residence time. Ethnobotanical data was collected from residents of the community through semi-structured interviews in January/December/2016. Species diversity was calculated using Shannon-Wiener, Level of Fidelity (LF), Correction Factor, and the Percentage of Agreement regarding the Main Uses (AMU). Statistical tests were performed using generalized linear models (GLM) in the R environment. The plant use indications were grouped according to the International Classification of Diseases and Related Health Problems (ICD 10). We found 152 species belonging to 130 genera and 67 families. The most frequently used plant parts were leaves, and decoction was the most frequent preparation mode. *Strychnos pseudoquina* was the species with the highest amount of use indications. The diversity index was 4.5 nats/ind^-1^. The body system with the most citations was the code XVIII of ICD 10, corresponding to the species: alfavaca, mentraste, terramicina, angelim, fedegoso. Medicinal species with AMU values higher than 25% were: *Strychnos pseudoquina*, *Plectranthus barbatus*, *Citrus sinensis* cv. *pera*, *Cymbopogon citratus*. There was a relationship between the number of useful plants and the residence time of the participants. The Bananal community revealed high species richness and the relationship of knowledge showed that the older the residents and the longer their residence time in the community, the more knowledge they acquired.

## Introduction

Ethnobotanical studies have been carried out throughout human history. Such studies started off as qualitative descriptions, but later on focused on quantitative analyses that evaluated levels of fidelity and agreement of use of the species, and now used as statistics with inferencial analyses. Quantitatively, it is possible to demonstrate the importance of different plant characteristics by investigating the knowledge, uses of such plants within a society [1], and practical applications for intercultural comparisons since such method allow for a consensus about knowledge variations [2].

In developed countries, there are less ethnobotanical studies in comparison to developing countries. Some recent works include studies from Italy [3,4], from Iberian Peninsula countries [5] and from France [6]. In the latter, the research focused on plants that are used to produce cosmetics and perfumes. In developing countries, more ethnobotanical research is done, being superior and displaying that plants are still widely used among human populations, and oftentimes are one of the few available resources for disease treatment. Such works, which evaluate traditional knowledge, are frequently published in countries as Kenya [7], Ethiopia [8], Angola [9], Ecuador, Peru, Bolivia [10, 11], among others. Besides these, other studies have been accomplished in distant places that are difficult to access, as an area in the Himalayan mountains [12], a district situated in South-Eastern Bangladesh [13], and an area on the northwestern coast of Egypt [14].

In light of these surveys, ethnobotanical studies are important throughout the world because of communities that use plant resources for subsistencc. Dissemination of such data favors the exchange of knowledge between communities from different places in the world, forming a knowledge network. Currently in Brazil, most of the population still uses medicinal plants to alleviate or even cure certain diseases due to their low cost and/or effective results [15–18]. The diversity and availability of native plants favor their use by diverse peoples and communities [18], such as rural (non-traditional) communities, traditional communities, indigenous populations, quilombolas, riverside. The rural communities (focus of the present study) are able to identify many plant species that generate several products, including food, firewood, medicine, fodder and tools for their daily necessities. Thus, ethnobotanical studies help assess how residents in a community mix previous knowledge of their homelands with information from new settlements. Since these people come from various regions of Brazil, they need to adapt and acquire useful plants in their new environments [19].

Such knowledge about useful plants is only orally passed down from one generation to the next [17, 18, 20, 21] and in rural communities is limited to only certain community members, and is gradually decreasing [18]. Many people in rural communities have ample knowledge about these plants, as well as usage methods, practices, and beliefs [18, 22]. Thus, ethnobotanical studies are urgently needed to document current knowledge and provide a baseline for future analysis regarding knowledge and use of native plants [12, 23]. Thus, observing such line of thought, we raise the following hypotheses: rural communities use a wide variety of plant species; there is a consensus among community informants regarding the use fidelity and main use of plant species; there is a proportional relationship between older informants, residence time in the community, and education with more knowledge of the species used.

This study aimed to: (i) determine the plant species used by the rural community of Bananal, Mato Grosso, Brazil, (ii) verify the levels of fidelity and agreement of uses among the local inhabitants, (iii) determine the relationship between the number of known medicinal plants and the levels of education, age, and residence time.

## Methodology

### Description of the study area, data collection and botanical identification

**Fig 1:**
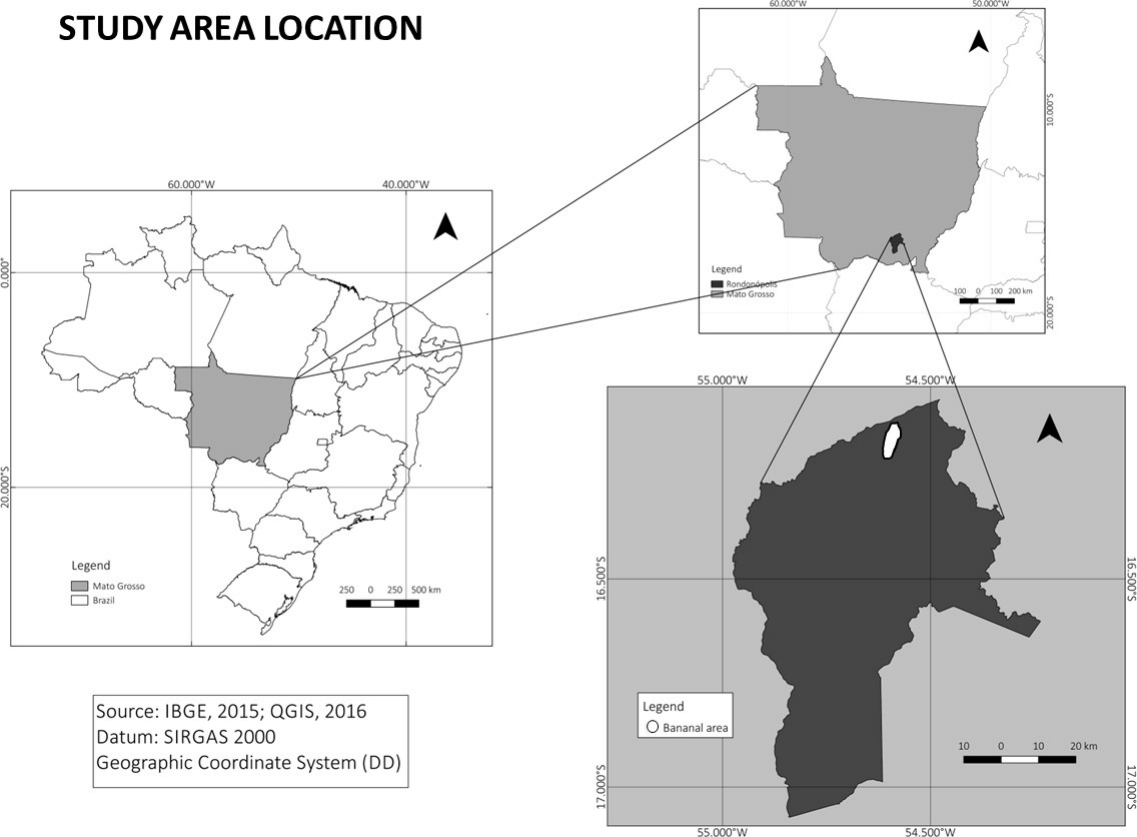
Geographic location of the study area, Bananal Community, Rondonópolis, MT.

The study was conducted in a rural area in the Bananal Community, located in the northern region of the city of Rondonópolis, Mato Grosso, Brazil, at the coordinates 16°8’58.68”S and 54°35’10.63”W (Fig 1). The community is made up of the Bananal and Olga Benário settlements that are linked to health care in the Family Health Strategy (FHS) Unit of Bananal. The choice of the place of study was conducted randomly by lottery among the FHS from rural areas.

According to Köppen, the local climate is Tropical Aw. The average annual temperature is 27°C, with a maximum of 40°C in September and a minimum of 9°C in July. The highest average rainfall occurs in January, with 320mm, and the lowest in June [24].

The vegetation is divided into regions of gallery forests, savannas, and grasslands, and presents a transition between savanna woodland (Cerradão) and riparian forest. The range of riparian forest vegetation runs parallel to the watercourse and represents the largest part of the remnant native vegetation. The areas of Cerrado present woody vegetation with trees, shrubs, and native herbaceous plants.

Historically, the colonization of the Bananal region began around 1920, with the migration and occupation of landless workers in the Rondonópolis territory [25]. In the Bananal community, the economy presents significant agricultural potential, with prevalent perennial and annual crop cultivation for both consumption and sale. Cattle and pig livestock are raised with emphasis on milk and cheese production and sales, while beef cattle are raised only for local consumption. The region receives services ranging from energy and water supply to bridge and road maintenance.

The community is assisted by programs that provide courses, lectures, and workshops that invest in quality of life and the implementation of family agriculture, such as ponds for fish farming, spring recovery, guidance for field activities, hydroponics implementation, as well as vegetable seeds for families interested in growing vegetable crops [26].

After the determination of the study site, contact was made with the nurse and Community Health Agent (CHA), who are members of the FHS care team. The research was explained and invited to CHA to accompany the researcher in recognizing the area and visiting the homes of people in the rural community, since the residences are far from each other. Follow-up by a local representative helped establish a good relationship between researcher and community residents. They were informed and invited to participate in the survey. Data collection was between January and December 2016, which consisted of semi-structured interviews in the participants' homes.

The following requirements were established for all research interviews: prior consent, availability of participant, all participants were 18 years old or older, participants were residents and registered by the FHS, and family member(s) who had knowledge of useful plants for any purpose. The study excluded residents who were not found after three attempts during the data collection period. The study was carried out with 50 residents from the rural community, the interviews were previously authorized by all the respondents, who signed a Term of Free and Informed Consent, and the CHA as the local representative of Health in the Community, signed an authorization for research on behalf of the entire community.

The species of plants mentioned were defined as sampling units. The species were identified in the field and some collected and photographed for records and subsequent identification without the need of specific organs permissions, because they were on private land and have been authorized by the representative, not involving endangered or protected species. They were collected at several places in the following coordinates: place of collection 1: 16° 8'50.35"S and 54°34'58.64"W; place 2: 16° 8'57.66"S and 54°34'54.21"W; place 3: 16° 8'39.16"S and 54°34'59.71"W; place 4: 16° 7'24.03"S and 54°34'38.04"W; place 5: 16° 7'26.04"S and 54°34'49.02"W; place 6: 16° 7'16.10"S and 54°34'9.38"W; place 7: 16° 7'25.24"S and 54°34'2.24"W; place 8: 16° 7'49.98"S and 54°33'57.06"W.

The collected material was herbarized and identified by consulting specialized literature and botanical experts, as well as through comparison with exsiccates. Of these, the reproductive stages were deposited into the UFMT Herbarium at the Universidade Federal de Mato Grosso (UFMT), Cuiabá, MT.

The scientific names of the species were conferred using online databases of the Missouri Botanical Garden/Tropics [27] and the species list from the Flora do Brasil [28]. For taxonomic classification, we used the Angiosperm Phylogeny Group IV [29]. For geographical origin, we consulted the header of the Species List from the Flora do Brasil [28] and used the following classifications: native, naturalized, cultivated, and exotic.

The empirical classification was used to describe the use category of the cited species. The cited species were grouped into seven categories: medicinal, medicinal and food, food, medicinal and other (used to manufacture soap for general cleaning; used as a flavoring agent), ornamental and mystical, medicinal, food and lumber, and medicinal, ornamental, and mystical.

Plant preparations were classified as follows: decoction (plant part was cooked) as tea, molasses, syrup, and drink with toasted plant, infusion (plant part was put in hot water), ingestion (plant part was used in water or juice, including sap used in coffee, wine, or milk), bottled medicine, and others.

Each plant use indicated by the participants was grouped according to the International Classification of Diseases and Related Health Problems (ICD 10), published by the World Health Organization [30].

### Statistical analyses

To verify species diversity, we used the Shannon-Wiener index (H’) applied to ethnobotany as described by [31], which is represented by the following formula:

$$H'=\sum_{i=1}^{S}\left(\mathrm{pi})(\log2\;\mathrm{pi}\right)$$

Where: H’ = Shannon-Wiener diversity index using log base *e* (*nats*/individual)

S = Number of species

pi = Relative proportion of abundance of species *I* in relation to the total number of species cited.

Later, the Pielou evenness or uniformity measure was used to measure the distribution pattern in the community [31]. This calculation is expressed by the following formula:

$$J'=\frac{H"}{H"\;\mathrm{Max}}$$

Where: J’ = Shannon-Wiener function Evenness measure (varies from 0 to 1)

H’ = Shannon-Wiener diversity index

H’ _Max_ = Maximum value of H’

One technique we used to analyze the relative importance of a plant in the community was the consensus among participants. The consensus analysis was based on the concordance between participant responses.For each plant, we also calculated an index called Level of Fidelity (LF) [31,32], expressed by the following equation:

$$\mathrm{LF}=\frac{\mathrm{Ip}}{\mathrm{Iu}}\times 100$$

Where: LF = level of fidelity

Ip = number of informers that mentioned the main use of a particular species.

Iu = number of participants that mentioned the species for any purpose.

The Correction Factor (CF) was estimated to obtain consensus among the informants for each plant. Its value is derived from the number of citations of a certain species, i.e., the number of participants that mentioned each species divided by the number of citations for the most commonly mentioned species, i.e., the number of participants that mentioned the most commonly mentioned species.

Calculating the CF:

$$\mathrm{CF}=\frac{\mathrm{Iu}}{\mathrm{number}\;\mathrm{of}\;\mathrm{informers}\;\mathrm{that}\;\mathrm{mentioned}\;\mathrm{the}\;\mathrm{species}\;\mathrm{more}\;\mathrm{mentioned}}$$

Then, the percentage of Agreement regarding the Main Uses (AMU) was calculated to neutralize the higher or lower popularity of the species. This calculation is based on the value found in the LF multiplied by the correction factor, expressed in the following formula:

AMU=LF×CF

This analysis can be adapted to any use category, but was initially used in studies on medicinal plants [33]. In this sense, we decided to keep the analysis only to measure the relative importance of plants indicated as medicinal due to the high number of citations for this category.

Statistical models were used to explore how social and cultural variables interact with each other and with the knowledge of plant collection, use and management intensity [2]. We use the methodology described by [2], where the socioeconomic and demographic data were grouped into categorical variables (gender, education) and the continuous variables (age and residence time). For the variables, a generalized linear model with negative binominal distribution was used to see the effect of residence and age and experience of participants in the number of used species. All analyses were performed in R [34].

### Ethical aspects

The research project was sent to the Committee of Ethics in Research with Human Beings of the University Hospital Julio Muller (CER / HUJM), Cuiabá / Mato Grosso, in compliance with Resolution 466, of December 12, 2012, of the National Health Council. The CER / HUJM approved all aspects of the research and issued a favorable opinion for the development under the number of CAAE: 48675315.0.0000.5541.

The research in rural community was provided by the Municipal Health Department of Rondonópolis, in Mato Grosso. In the interview, participants were informed about the research and their participation on a voluntary basis, they signed the Informed Consent Form authorizing the participation. In addition, a local representative, resident in the Community, also signed an authorization to conduct the survey on behalf of all, including the collection of some species for botanical identification on their properties, without the need for permissions of specific organs.

## Results and discussion

Of the 50 interviewees, 28 were males and 22 were females; age ranged from 34 to 81, with a predominance of 50–59 year-olds. The most frequent level of education (34%) was incomplete elementary school, however, the educacional level of the interviewees varies from literate to higher with postgraduate studies. Family farming and retirement sustain the community. More than half of the interviewees (54%) were from the state of Mato Grosso.

In relation to plants, the participants mentioned 152 species of useful plants, distributed in 130 genera and 67 families (S1 Table). The number of plants we found was higher than other ethnobotanical studies, which registered values between 46 to 72 genera and 33 to 46 families [35, 36]. The present work stands out because of the high number of useful plants, however, this study was only developed in one community, while other studies were carried out in two or more communities [35].

**Fig 2:**
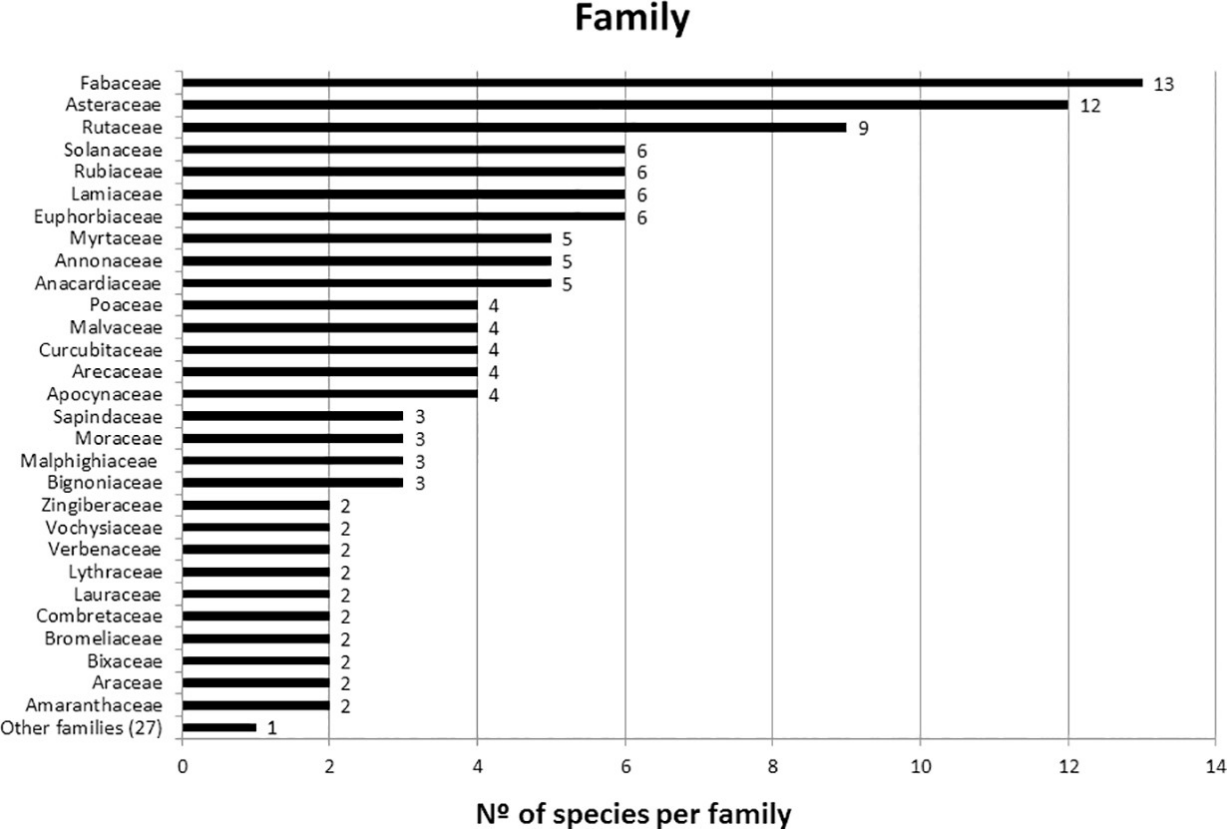
Species richness by family from interviews in the Bananal Community, MT, Brazil.

The botanical families with the highest number of useful species were Asteraceae (12), Fabaceae (11), Rutaceae (9), which represented 21% of all species recorded (Fig 2). Such results have commonly been found for these families from ethnobotanical studies in this region [37,38] and in other countries with indigenous peoples and traditional or rural communities [35,36,39–44]. This is due to the cosmopolitan distribution of these families, except Antarctica, which are spread across all continents of the world and all Brazilian regions [45–47].

This work evidences the ethnobotanical importance of the native Cerrado plant species and their relationship with the local inhabitants. Empirical knowledge of these species is high considering the number of species, and is capable of generating economic, social, and ecological values for these species. Such species are of fundamental importance for local development as a means of subsistence and immediate relief of health problems in the region, which clearly demonstrates the interaction between community and the environment.

Through the interviews, we obtained 1,070 indications of use for the species reported. These values showed high ethnobotanical knowledge of plants, especially for medicinal plants, in this region. Several studies have been developed aiming to collect information regarding the knowledge of native plants use by different ethnic groups, such as [11,44,48], in more specific groups, such as the [13, 49,50], Quilombolas [51] and in rural communities [2,44]. The results obtained in the present work provide a wealth of knowledge and a surprising number of species citations when compared to the studies mentioned above.

Among the 152 species, the most frequently mentioned were *Strychnos pseudoquina* A. St.–Hil (Loganiaceae) with 50 citations, followed by *Hymenaea stigonocarpa* Mart. Ex (Fabaceae) (47 cit.), *Citrus sinensis* Osbeck cv. *Pera* (Rutaceae) and *Plectranthus amboinicus* (Lour.) Spreng. (Lamiaceae) (29 cit.), and *Senna occidentalis* (Fabaceae) (L.) Link. (23 cit.) (S1 Table).

The species *Strychnos pseudoquina* (quina) was indicated for treatment of bodily problems, with several purposes including digestive, vermifuge, depurative, appetite stimulant, anemia, diabetes, cough, headache, however the most frequent citation was for problems with the digestive system (S1 Table). We found that such purpose corroborates with a study proving the gastro protective activity of *Strychnos pseudoquina* [*52*]. Several studies about *S*. *pseudoquina*, with pharmacological, ethnobotanical, and phytochemical approaches have been conducted and indicate anti-inflammatory, anti-herpes and anti-leishmaniasis healing properties [53–58].

*S*. *pseudoquina* is a native plant with distribution in South America, registered in Bolivia, Paraguay and Brazil [27], and in Brazil it is geographically distributed in the northern, northeastern, midwestern, and southeastern regions [28]. In addition, the species’ ecology favors its propagation, as it is fire tolerant and tends to proliferate in deforestation [59]. As this species is widely distributed in almost all Brazilian regions, including the study site, and is easily spread, it is frequently used and a widely available raw material.

*Hymenaea stigonocarpa* (jatobá) is distributed in South America in countries such as Brazil, Bolivia, Paraguay and Venezuela [27] and is a species relevant to the Bananal community, featuring 47 use citations, including timber, medicinal, treatment for bodily diseases such as pneumonia, flu, bronchitis, cough, anemia, depurative, stomach problems, respiratory problems, anti-inflammatory, cancer treatment, and as a food item (S1 Table). The plants of this genus present bioactive compounds, which confirms the effectiveness of some traditional medicine indications [60]. There are several scientific works that indicate gastroprotective activity, phytochemical and antimicrobial activity, anti-inflammatory and antioxidant action, as well as antidiarrheal and healing properties for duodenal and gastric ulcers [61–64]. However, a recent study about the genus *Hymenaea* spp. tested commercial sap samples and detected adulteration and microbiological contamination, which could be a risk to human health [65]. Thus, we emphasize the importance of the origin of the product.

Regarding the origins of the 152 identified useful species, 73% were native species and 27% were naturalized, cultivated or exotic. However, in relation to use indication, cultivated species stood out because of their availability and ease of collection, as they were often present in backyards or in vessels near homes. Among the cultivated and exotic species found in the community, we cite *Curcuma longa* L. (acafrão) with European origin, *Aloe vera* L. Burm.f. (babosa) from Africa, *Cymbopogon citratus* (DC.) Stapf (capim-cidreira) from Asia, *Punica granatum* L. (romã) from the Mediterranean, and *Ocimum basilicum* L. (alfavaca) from India (S1 Table**)**.

### Species diversity and relative importance

The species diversity was H’ = 4.5 nats individual^-1^ and species evenness was J’ = 0.9. Such high diversity values were also found in other studies [66–68]. Diversity was based on the knowledge regarding plant use of participants and this high value indicates that the interviewees mentioned a significant number of useful species. Overall, this suggests that residents have vast knowledge about plant use, since this diversity index [31] increased with the number of species in the community.

Of the 152 useful plant species recorded, 136 were indicated for medicinal purposes. Of these, the Agreement regarding the Main Uses (AMU) revealed four species with values higher than 25%, representing only 2.9% of the useful species. These species were *Strychnos pseudoquina* (50%), *Plectranthus barbatus* Andr. (40%), both mainly used for digestive problems, *Citrus sinensis* cv. *pera* (28%), primarily used for the flu, and *Cymbopogon citratus* (DC.) Stapf (26%), primarily used as a calmative (Table 1).

**Table 1:** Estimation of Level of Fidelity (LF), Correction Factor (FC) and Agreement regarding Main Uses (AMU) for each useful species with medicinal purposes in the Bananal Community, Rondonópolis, MT, Brazil. AMU% values in decreasing order.

Species	IP	IU	Main Use	LF	CF	AMU%
*Strychnos pseudoquina*	25	50	Digestive	50.00	1.00	50.00
*Plectranthus barbatus*	20	20	Digestive	100.00	0.40	40.00
*Citrus sinensis* cv. pera	14	21	Flu	66.67	0.42	28.00
*Cymbopogon citratus*	13	18	Calmative	72.22	0.36	26.00
*Plectranthus amboinicus*	12	21	Flu	57.14	0.42	24.00
*Terminalia fagifolia*	11	16	Stomach problems	68.75	0.32	22.00
*Mentha spicata*	10	13	Flu	76.92	0.26	20.00
*Lippia alba*	10	17	Calmative	58.82	0.34	20.00
*Mentha pulegium*	10	16	Flu	62.50	0.32	20.00
*Ocimum basilicum*	10	18	Flu	55.56	0.36	20.00
*Hymenaea stigonocarpa*	10	42	Flu	23.81	0.84	20.00
*Palicourea xanthophylla*	8	13	Kidney problem	61.54	0.26	16.00
*Citrus* x *limon*	8	15	Flu	53.33	0.30	16.00
*Vatairea macrocarpa*	8	19	Stomach problems	42.11	0.38	16.00
*Pterodon emarginatus*	7	14	Throat infection	50.00	0.28	14.00
*Senna occidentalis*	7	23	Flu	30.43	0.46	14.00
*Jacaranda rufa*	7	11	Depurative	63.64	0.22	14.00
*Brosimum gaudichaudii*	6	15	Depurative / vitiligo	40.00	0.30	12.00
*Dysphania ambrosioides*	6	18	Vermifuge	33.33	0.36	12.00
*Croton urucurana*	5	17	Infection	29.41	0.34	10.00
*Alternanthera brasiliana*	5	12	Anti-inflammatory	41.67	0.24	10.00
*Lafoensia pacari*	5	19	Skin disorders	26.32	0.38	10.00
*Stryphnodendron adstringens*	5	15	Infection	33.33	0.30	10.00
*Tabebuia aurea*	4	11	Vermifuge	36.36	0.22	8.00
*Ruta graveolens*	4	17	Headache	23.53	0.34	8.00
*Aloe vera*	4	22	Burn / câncer treatment	18.18	0.44	8.00
*Chiococca alba*	3	14	Headache / snake bite	21.43	0.28	6.00
*Jatropha elliptica*	3	14	Depurative / vermifuge	21.43	0.28	6.00
*Momordica charantia*	3	11	Stomach problems / câncer treatment	27.27	0.22	6.00

IP = Number of people that mentioned the species for the main use; IU = Number of people that mentioned any species use.

This study highlightsthe species *Strychnos pseudoquina*, as all participants mentioned that it had at least one use and half of the participants indicated it for the same purpose. This reflects the species’ popularity and the knowledge the community has in relation to its therapeutic actions, with several studies [52–58] demonstrating the pharmacological actions of *S*. *pseudoquina*.

Additionally, although *Plectranthus barbatus* was only indicated for digestive problems by participants, a review of the species [69] mentions pharmacological actions, including hypotensive, positive-inotropic actions, cardiovascular, bronchodilator, activation of adelilatocyclase, inhibition of platelet aggregation (anti-metastasis), antitumor, anti-inflammatory and anti-nociceptive. Another study [70] identified in vitro anti-transpanosomal activity and a brief literature review revealed it has antioxidant, antimicrobial, antifungal, and anti-acetylcholinesterase activities. Furthermore, another study [71] referred to this species’ potential antimalarial action.

A large proportion of species (97.5%) presented low AMU% (below 25%). However, this does not mean these species are irrelevant to the community, which is possibly related to the wide range of indication of species reported by informants, but few useful purposes. Even though the Bananal community expressesed species diversity, their use citations were restricted.

### Use categories, lant parts used and reparation mode

According to participant indications, use categories were: medicinal, medicinal and food, food, medicinal and other (soap manufacturing, flavoring), ornamental and mystical, medicinal, food and lumber, and medicinal, ornamental and mystical (S1 Table). Among these, plants with exclusive medicinal use were the most representative, with a total of 68%. Correspondingly, the medicinal category is frequently mentioned in other research articles, reaffirming the diversity and availability of plant resources with medicinal potential [72–76]. Therefore, it is clear that the Bananal community has been using plants to treat and cure their illnesses and diseases, as they are predisposed to difficult access to health centers and high costs of allopathic medicine [21,77], as well as their cultural legacy.

Essentially, the population primarily uses medicinal plants, however, such plants may have more than one use purpose. We recorded multipurpose (used for different purposes) plant species as *Hymenaea stigonocarpa*, which was mentioned as a wood source, food item, and for its medicinal purposes. Thus, 18.4% of the mentioned species had medicinal and food purposes and 9.2% were exclusively used for food industries (S1 Table).

In the present study, food plants use was low. According to [78], food purposes are not very common, which can lead to the abandonment or knowledge loss for these plants in communities. In addition, it is important to motivate rural communities to consume native edible species, which can even help increase family incomes [79]. Another type of use was mystical-religious purposes. Species with such purposes were found in some homes and represented ways of acting and thinking for the community, confirming practices involving beliefs and spirituality. Plants, such as *Dieffenbachia seguine* (Jacq.) Schot (comigo-ninguém-pode) and *Ruta graveolens* L. (arruda) were claimed to protect the home and ward off evil, demonstrating the symbolic-mystical-religious of the local people [80].

**Fig 3:**
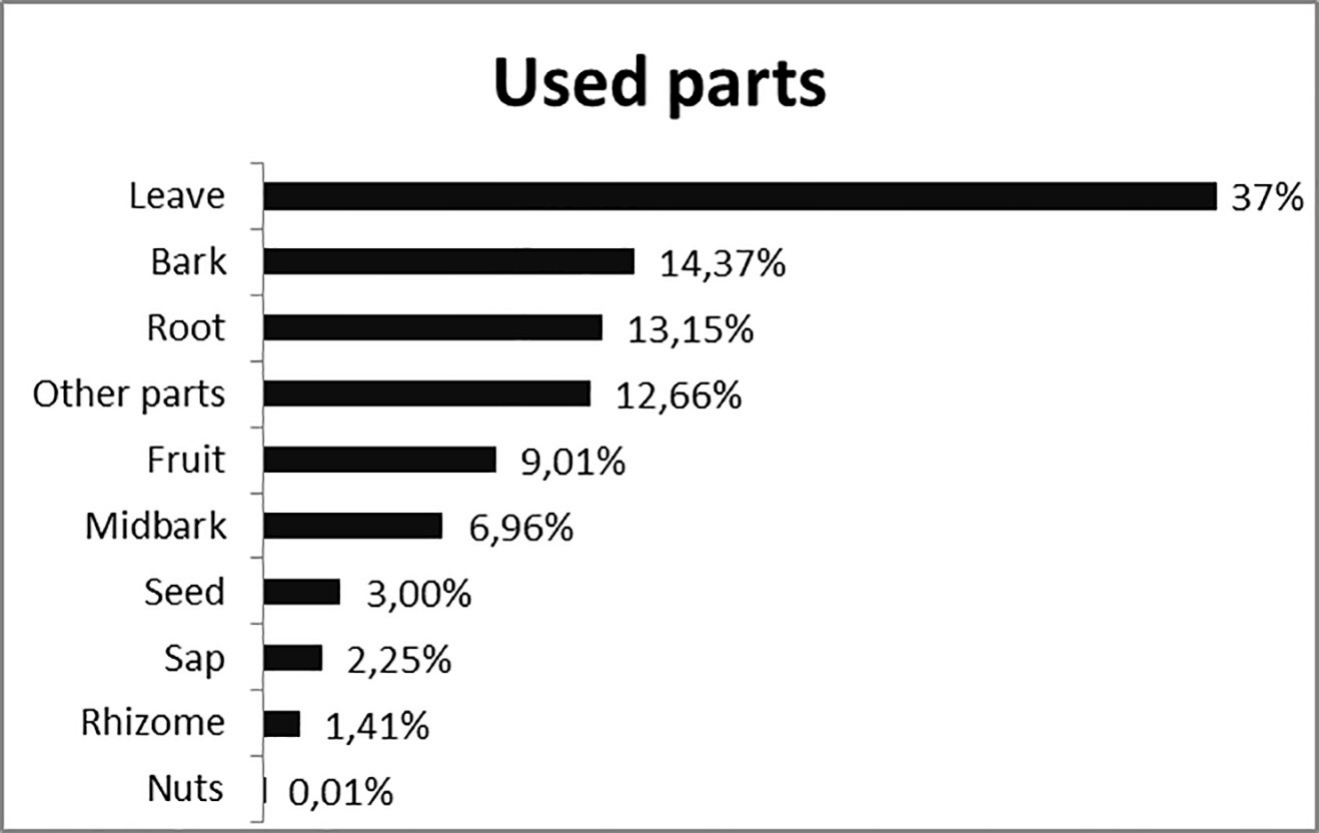
Plant parts used by the Bananal Community, Rondonópolis, MT, Brazil. Other parts: flower, mesocarp, bud (leaf apex), heartwood, oil, pulp, branch, bud.

Regarding plant parts used to prepare medications, the most prominent parts were leaves (37.1%), bark (14.3%) and roots (13.1%) (Fig 3). Such data agrees with other works that recorded 36.4% and 64.5% for leaves, and 23.7% and 7.8% for barks [37, 81].

The leaves were the most frequently mentioned parts in other studies with 72.5%, 68.2%, 61%, 40%, respectively [82,67,83,84]. This may be due to the fact that leaves are the most abundant and accessible plant parts [81]. From the conservation point of view, the use of leaves is sustainable, since, if the withdrawal of aerial parts is not excessive, will not prevent the development and/or reproduction of the plant [85].

**Fig 4:**
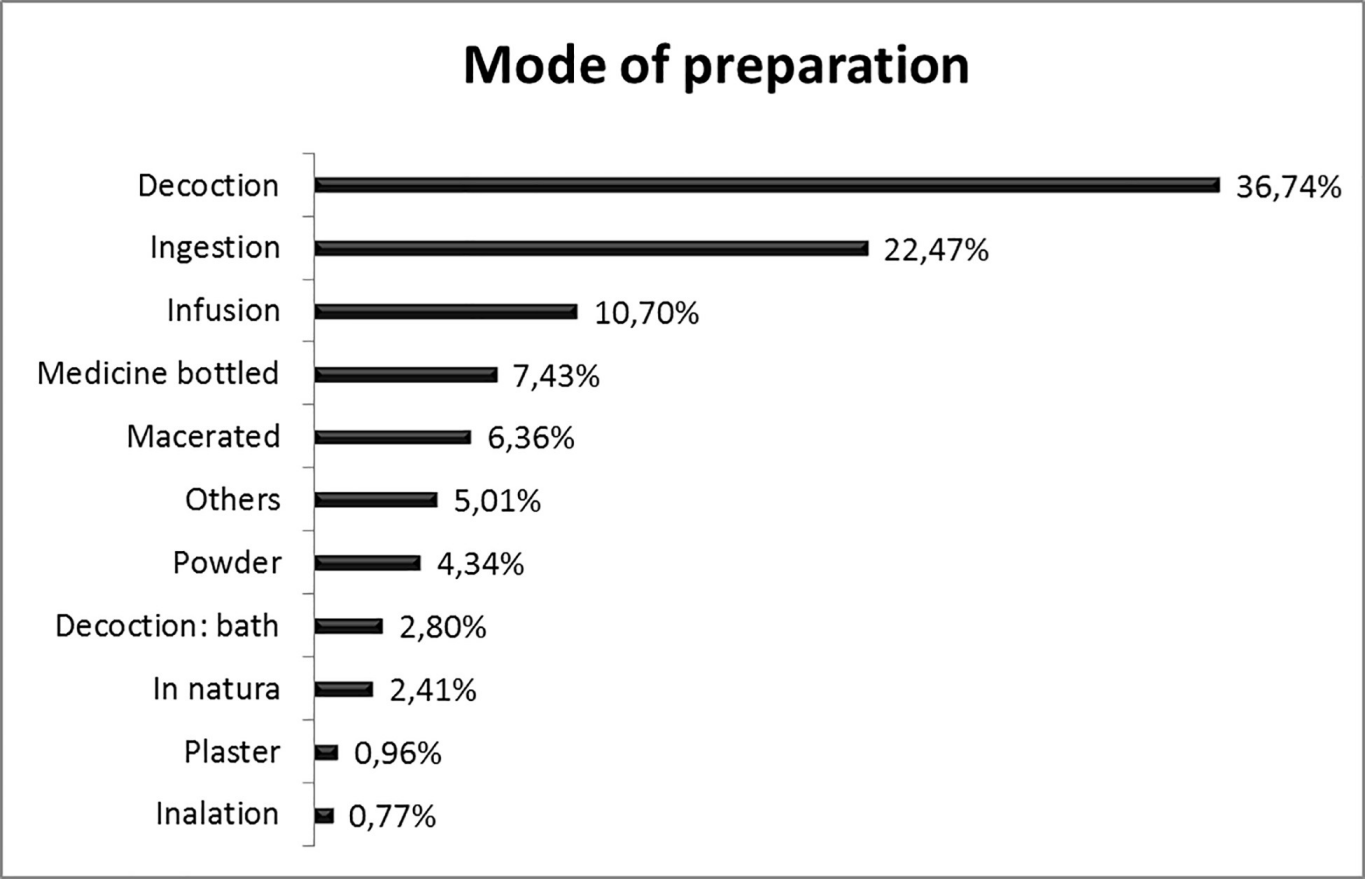
Preparation mode of useful plants by the Bananal Community, Rondonópolis, MT, Brazil. Other: topical use (powder, ointment, oil, *in natura* on the area), eye wash, mouthwash, gargle. Note: Decoction for bath: bath, seat bath, wash wound.

The research participants reported several preparation modes for the species with medicinal purposes. Decoction was the most common preparation mode (36.7%), followed by ingestion (22.4%), infusion (10.7%), and bottling (7.4%) (Fig 4).

Decoction was also the most frequently used method by people in other communities [35,44,42,86]. How people make homemade extractions with active components is important and should be taken into consideration, since the concentration of active substances increases or decreases depending on the production mode [87].

The popular knowledge in the Bananal community is vast, as the participants reported that harvesting, as well as preparation and measuring, of plant material should be done carefully. They also mentioned combining some herbs to obtain more effective results, as *Digitaria insularis* (L.) Fedde (capim-amargoso) added to *Momordica charantia* L. (melão-de-são-caetano) to treat renal infections. The practice of combining one or more plant species was expressed in other studies [88,89] and provides synergistic pharmacological actions between plants [84].

Popular recipes depend heavily on the availability of local plants [90]. Knowledge about the plant action, the availability of raw materials, access to the plant, how quickly they can be used, and their immediate side effects, demonstrate the importance of these plants for the health care of the people in a community. Furthermore, participants indicated that we should be careful when using certain medicinal plants, as they can cause adverse reactions. From a scientific point of view, many of the plant species have potentially aggressive substances and should be used with care, respecting their toxicological risks [91], as well as the practice of replacing allopathic medication with plants and concomitant use, since they also present risks to the population [92]. In that case, medicinal plants can aid in a healthy recovery, but should not be used indiscriminately or without knowledge of their therapeutic action.

### Therapeutic indications/purpose–ICD 10

Grouping by ICD-10 serves to inform the classification code of diseases and a wide variety of signs, symptoms, abnormal aspects, complaints, social circumstances, and external causes for injury or illnesses. Each state of health receives a bodily category or system, which corresponds to a code represented by roman numerals from I to XXII. This code is often used for classifying diseases. In this study, the classification was performed with the objective of evaluating the most affected diseases and body systems in the community.

**Fig 5:**
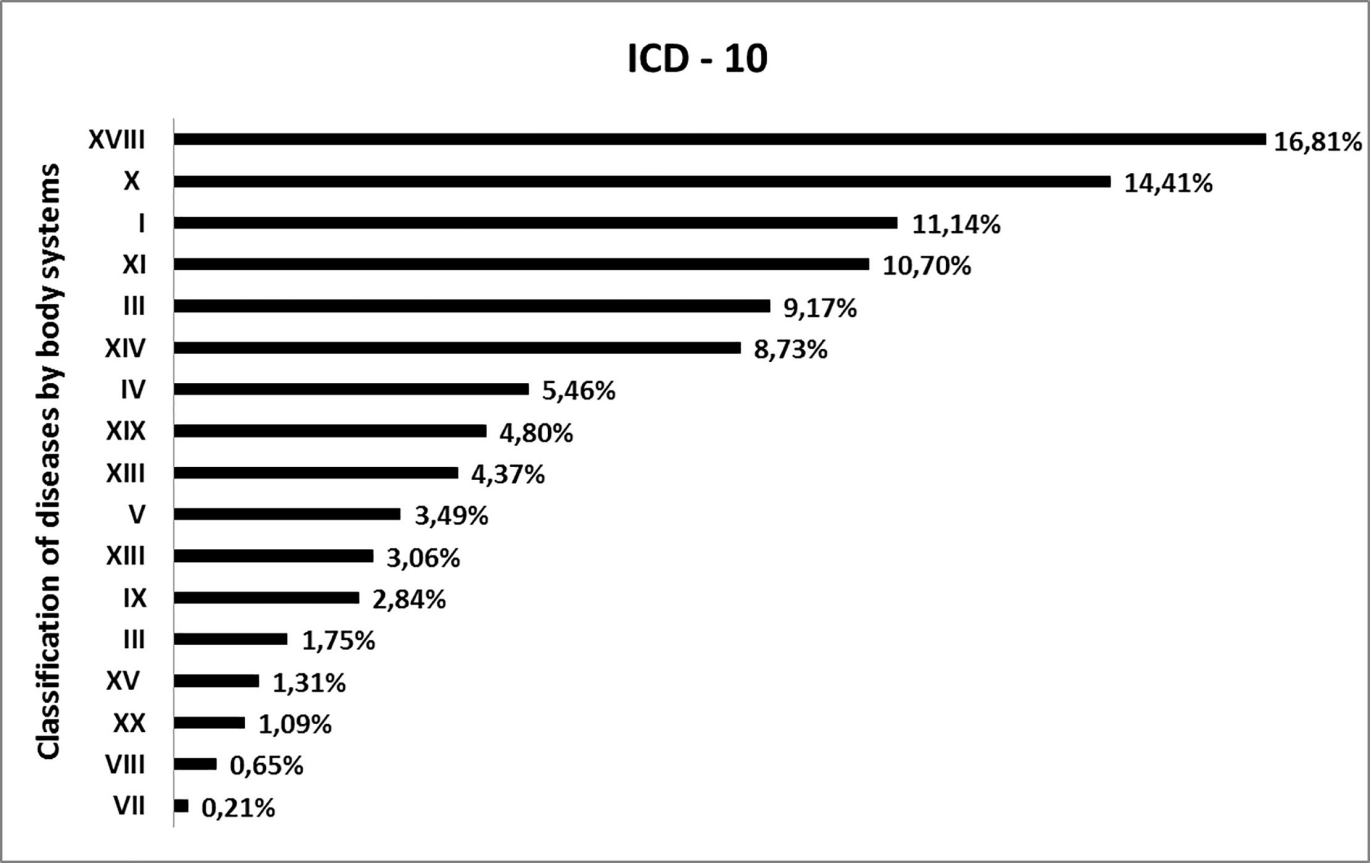
Citation frequency of medicinal plant used to treat bodily systems, classified according to ICD-10, by the participantsof the Bananal Community, Rondonópolis, MT, Brazil. Subtitle: **I** Infectious and parasitic diseases, **II** Neoplasms (tumors), **III** Blood and hematopoietic organ diseases and some immune disorders, **IV** Endocrine, nutritional and metabolic diseases. **V** Mental and behavioral disorders, **VII** Diseases of the eye and attachments, **VIII** Ear and mastoid disorders, **IX** Diseases of the circulatory system, **X** Diseases of the respiratory tract, **XI** Diseases of the digestive system, **XII** Skin and subcutaneous tissue disorders, **XIII** Diseases of the musculoskeletal system and connective tissue, **XIV** Diseases of the genitourinary system, **XV** Pregnancy, childbirth and the puerperium, **XVIII** Symptoms, signs, and abnormal findings of clinical and laboratory exams, not elsewhere classified, **XIX** Injuries, poisonings and some other consequences of external causes, **XX** External causes of morbidity and mortality.

The therapeutic indications of the136 medicinal species mentioned by the Bananal community were categorized according to the ICD-10. After organizing this information, we found that plants were indicated for 17 of the 22 bodily systems of ICD-10. The most frequent citation was code XVIII, with 16.8% (Fig 5), referring to the treatment of symptoms, signs, and abnormal findings of clinical examinations and laboratory tests, not classified elsewhere, with the main species: *Ocimum basilicum* L. (alfavaca), *Angeratum conyzoides* L. (mentraste), *Alternanthera brasiliana* (L.) Kuntze (terramicina), *Vatairea macrocarpa* (Benth.) Ducke (angelim), *Senna occidentalis* (L.) Link. (fedegoso). Following, code X represented 14.4% of citations and referred to diseases of the respiratory system, with the species: *Anadenanthera peregrina* (L.) Speg. (angico), *Attalea phalerata* Mart. ex. Spreng. (bacuri), *Senna occidentalis (*fedegoso) and *Hymenaea stigonocarpa* (jatobá). Code I (11.1% of citations) refers to infectious and parasitic diseases, represented by species: *Dysphania ambrosioides* (L.) Mosyakin & Clemants (erva-de-santa-maria), *Momordica charantia* L. (melão-de-são-caetano), *Terminalia fagifolia* Mart. (mussambé), and *Leptolobium dasycarpum* Vogel (unha-danta). Code XI (10.7% of citation) is related to diseases of the digestive system (Fig 5), and the species are: *Vatairea macrocarpa* (angelim), *Gymnanthemum amygdalinum* (Delile) Sch. Bip. ex Walp. (caferana) and *Leptolobium dasycarpum* Vogel (unha-danta).

The high citation value for code XVIII may be caused by commonly occurring diseases and diseases with unknown causes (Table 2). In places far from medical centers, medicinal plants are the first option for treating the initial signs and symptoms of a disease [93]. The results of this study are important to verify the diseases treated by native plants in this community and are similar to other studies that also point out the presence of the X and XI codes (Fig 5) [88, 94, 83, 67, 87, 95].

**Table 2:** Diseases treated by Bananal Community classified according to the ICD-10 Code XIII, Rondonópolis, MT, Brazil.

Code XVIII Diseases		
Baby colic	Gangrene	Neck pain
Children's flatulence	Headache	Pain
Cough	Headache	Pain in the body
Cramp	Hemorrhage	Swelling
Fever	Inflammation	Swelling in the feet
Flatulence	Jaundice	

#### Relationship of participant’s knowledge about useful plants

When verifying relationships of community knowledge about useful plants, inferential statistical tests showed that 73.1% of the use indications corresponded to 20 participants and approximately 26.9% of use indications to 30 participants. We infer that the community knowledge about useful plants was concentrated in a few residents of the community.

The number of years that participants resided in the study community was positively associated with the ethnobotany knowledge of plant use, with *P value* = p ≤ 0.001 (Table 3). A similar result was recorded in two indigenous communities [2], who reported that participants that lived in the study area for many generations possessed the highest knowledge of plant use, in comparation to those who recently moved to the communities.

**Table 3:** Generalized linear models showing the relationships between useful plant indications with residence time, age, education and the interaction between them. The type of distribution applied is listed below the name of the respective dependent variable (indication). The first numbers indicate the probability and the numbers in parentheses indicate estimation. Where: indication = number of useful plants mentioned by the participant.

Akaike’s Information Criterion (AIC)		Negative binomial (0)
**Intercept**	5.39e-06 *** (3.6905167)	
**Residence time**	0.01545 * (-0.0848917)	
**Education**	**0.292 (0.129575)**	
**Age**	**0.31020 (-0.0146152)**	
**Residence time x education**	**0.735 (-0.001143)**	
**Residence time x Age**	0.00828 ** (0.0015042)	
**Age x Education**	**0.791 (0.002158)**	
**Observations(n)**	**50**	

Codes meaning: 0 ‘***’ 0.001 ‘**’ 0.01 ‘*’ 0.05 ‘.’ 0.1 ‘‘ 1

**Fig 6:**
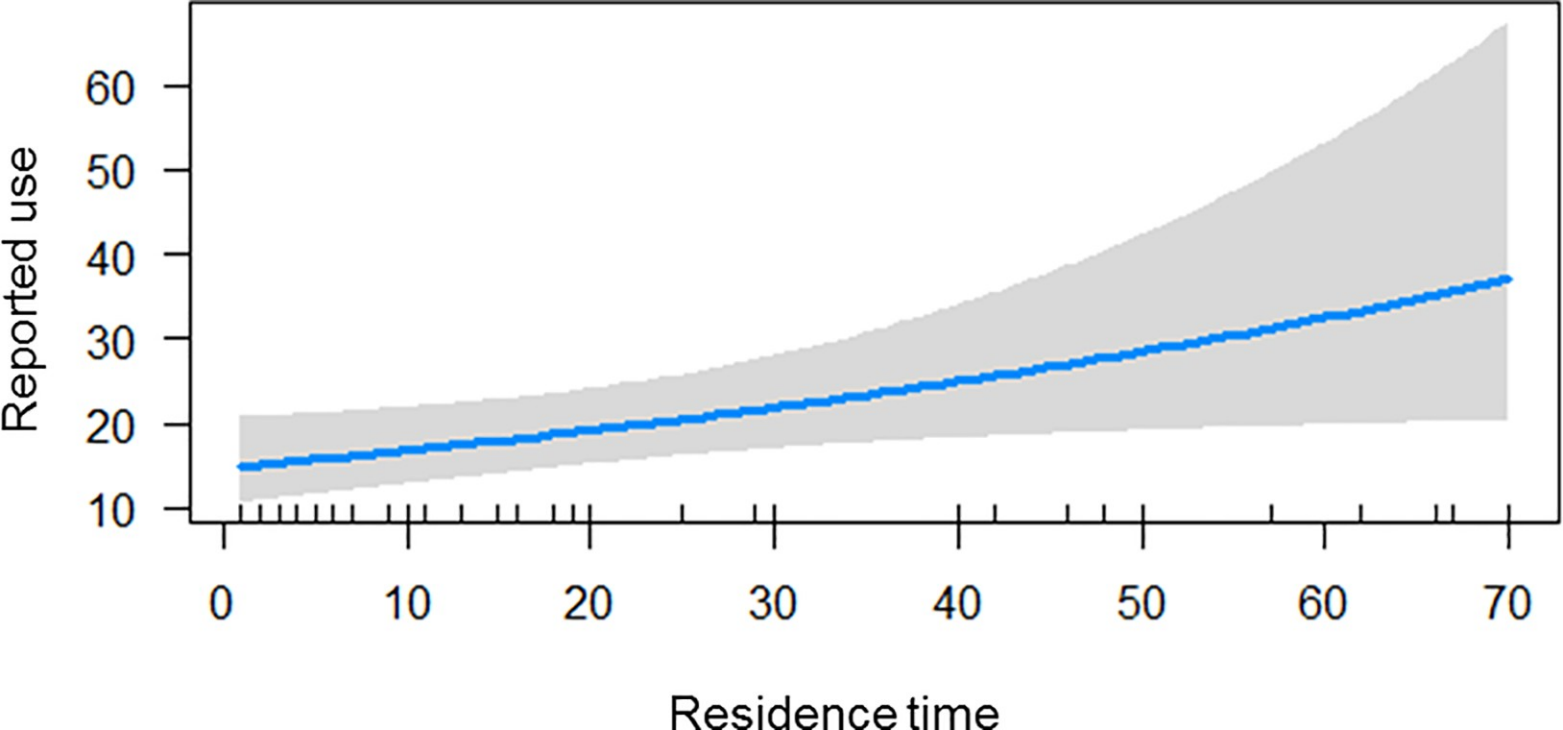
Generalized linear model of relationship between the dependent variable (use indication) and factor (residence time) in the Bananal community, Rondonópolis, MT, Brazil. 2018. Residence time is in (years), solid lines are real function and shaded areas are confidence intervals.

**Fig 7:**
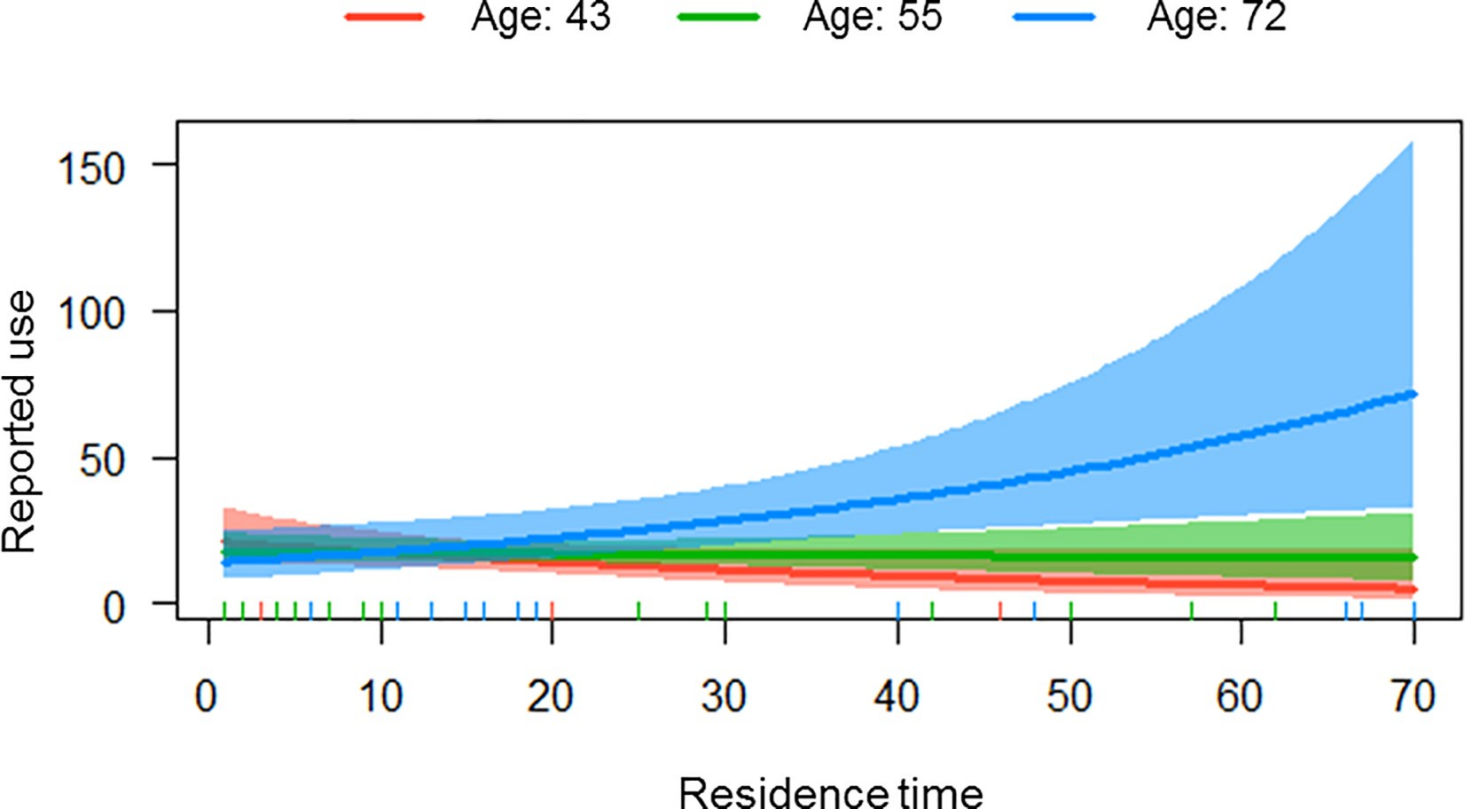
Generalized linear model of relationship between the dependent variable (use indication) and interaction of factors: residence time and age in the Bananal community, Rondonópolis, MT, Brazil. 2018. Residence time is in (years), solid lines are real function and shaded areas are confidence intervals.

Significant relationships appeared when the residence time in the community was analyzed individually (Table 3, Fig 6) and in the interaction between time and age (Table 3, Fig 7). In other words, the older the residents were, the more knowledge they had about plants. Likewise, the longer a resident lived in the community, the more knowledge they acquired about plant use.

Although the ages 43 to 55 years old were poorly represented, these variations did not affect our predictions, since more advanced ages (55 to 70 years) reinforced the interaction between residence time and age. Another study found different results, where people of different age categories (juveniles and adults) showed comparable knowledge of medicinal plants [96]. Thus, in the present study the interaction of residence time and age of each participant was an important factor foracquiring knowledge about the useful plants from the local flora. The older the participant and the longer their residence time, the greater their knowledge and indications of useful plants in this community.

Such results support that knowledge is concentrated in the older community members, since they indicated a higher amount of plant uses, and the youngest people had less knowledge of plant uses. Such lack of information may be due to young people’s lack of interest in useful plants species and may be influenced by increased technology. Furthermore, such useful knowledge, that is concentrated with the older members of the community, should be shared with other generations, as it could be lost if not passed on. Corroborating with our findings, [36,97] highlight that modernization and exposure to new drugs have significantly affected traditional practices, and ethnomedicinal knowledge is gradually being lost since older members are the main experts and the younger generation is not interested in learning this practice.

## Conclusion

This study significantly contributes to the value, socialization, and record of popular knowledge about useful plants, which was not being documented. This is the first record of popular uses of plant species in the Bananal rural community. This community revealed high species richness, with 152 useful plant species presenting values, including 136 medicinal plants. The Bananal community also presented 1,070 citations, with various use indications to treat health problems. Overall, the community showed high diversity of knowledge about the plant species, with a diversity index of 4.5 nats/ind^-1^. The preferentially used species were *Strychnos pseudoquina* and *Hymenaea stigonocarpa*. Our research showed that the older the participant was and the longer their residence time was, the more knowledge they had about useful plants. Such knowledge is concentrated in older community members. In this sense, encouraging knowledge socialization among the different generations is essential to ensure that beliefs, traditions, and culture are not lost over the years.

## Supporting information

S1 Table Plants used by the residentes in the Bananal Community, Rondonópolis MT. 2018.
(DOCX)
