# Supplementary material for: Plants used by the rural community of Bananal, Mato Grosso, Brazil: Aspects of popular knowledge
Source: PLoS One. 2019 Jan 30;14(1):e0210488. doi: 10.1371/journal.pone.0210488 (PMC6353550; doi:10.1371/journal.pone.0210488)
Supplement: S1 Table — 2018. (DOCX) [file pone.0210488.s001.docx]

**S1 Table -** Plants used by the residents in the Bananal Community, Rondonópolis , MT. 2017.

| **FAMILY/SPECIES** | **COMMON NAME** | **USAGE CATEGORY** | **USE PURPOSE** |
| --- | --- | --- | --- |
| **Adoxaceae** |  |  |  |
| *Sambucus nigra L.* | Sabugueiro | Medicinal | Anemia, cough, flu, stomach problems, measles, chicken pox (loose body lesions) |
| **Alismataceae** |  |  |  |
| *Echinodorus macrophyllus* (Kunth) Micheli | Chapéu de couro | Medicinal | Depurative, Anemia, Kidney problems |
| **Amaranthaceae** |  |  |  |
| *Dysphania ambrosioides* (L.) Mosyakin & Clemants | Erva de Santa Maria (mastruz) | Medicinal | Cough, influenza, vermifuge, Bruising, Antibiotic to treat bruise, Inflammation, gynecological infection, Anemia, Heart disease (wounds) |
| *Alternanthera brasiliana* (L.) Kuntze | Terramicina | Medicinal | Anti-inflammatory, Headache, Flu, Fever, Gynecological infection, Infection |
| **Anacardiaceae** |  |  |  |
| *Myracrodruon urudeuva* Allemão | Aroeira | Medicinal | Fracture Healing, Inflammation, Flu, Skin Wounds |
| *Spondias mombin* L. | Caja | Food | Food |
| *Anacardium occidentale* L. | Caju | Food | Food |
| *Anacardium humile* A.St.-Hil. | Cajuzinho do Cerrado | Food | Food |
| *Mangifera indica* L. | Manga | Medicinal, Food | Flu, cough, pneumonia, food |
| **Annonaceae** |  |  |  |
| *Annona reticulata* L. | Fruta do conde | Medicinal, Food | Diabetes, Kidney infection, food |
| *Annona muricata* L. | Graviola | Medicinal, Food | Cancer prevention, diabetes, food |
| *Annona squamosa* L. | Pinha | Food | Food |
| *Annona crassiflora* Mart. | Pinha do cerrado (Ariticum) | Medicinal, Food | Food, Infection, Snakebite, Cancer Prevention |
| *Duguetia furfuraceae* (A. St. Hill) | Sofre do rim quem quer (Pinha brava) | Medicinal | Kidney problem (Renal colic, kidney infection) |
| **Apiaceae** |  |  |  |
| *Pimpinella anisum* L. | Erva doce | Medicinal | Baby cramps, gas, calmative |
| **Apocynaceae** |  |  |  |
| *Mandevilla illustris* (Vell.) Woodson | Batata de Ipú (amaru leite) | Medicinal | Vermifuge, Rheumatism, Depurative |
| *Aspidosperma subincanum* Mart. | Guatambu | Medicinal | Diabetes |
| *Hancornia speciosa* Gomez | Mangaba branca (hancornia) | Medicinal, Food | Caimbra, Hypertension, Circulation, Stomach ulcer, Breast desinflammantion breastfeeding, Food |
| *Mandevilla velame* (A.St.-Hil.) Pichon | Velame branco | Medicinal | Vermifuge, Depurative, Rheumatism, Uric Acid, Grangrena (Nerve Disease) |
| **Araceae** |  |  |  |
| *Dieffenbachia seguine* (Jacq.) Schott | Comigo ninguém pode | Ornamental, Mystic | Ornamental and mystic (envy, evil expelling, etc.) |
| *Philodendron imbe* Schett. Ex endl | Imbé | Medicinal | Bursitis, Joint pain, Breast desinflammation after breastfeeding, spurs treatment |
| **Arecaceae** |  |  |  |
| *Attalea speciosa* Mart. ex Spreng. | Babacu | Medicinal, Food | Strengthening of bones, fortifying, weight loss, hypertension, food, construction |
| *Attalea phalerata* Mart. ex. Spreng. | Bacuri | Medicinal, Food | Cough, bronchitis, flu, food |
| *Cocos nucifera* L. | Coco | Food | Food |
| *Bactris setosa* Mart. | Tucum | Medicinal | Vascular problems |
| **Aristolochiaceae** |  |  |  |
| *Aristolochia claussenii Duch.* | Jarrinha | Medicinal | Shelter break (absence of lactation) |
| **Asparagaceae** |  |  |  |
| *Sansevieria trifasciata* Prain | Espada de São Jorge | Ornamental, Mystic | Ornamental and mystic (envy, evil expelling, etc ...) |
| **Asphodelaceae** |  |  |  |
| *Aloe vera* (L.) Burm. f. | Babosa | Medicinal, Cosmetics | Hair Treatment, Burn, Healing, Uterine Infection, Injury, Diabetes, Stomach Problems, Hemorrhoid, Cancer Prevention, Cancer Treatment |
| **Asteraceae** |  |  |  |
| *Acmella oleracea* (L.) R.K.Jansen | Agrião do mato (MT) Tucupi (AM) | Medicinal | Bronchitis |
| *Solidago chilensis* Meyen | Arnica | Medicinal | Bruising, Headache, Joint pain |
| *Vernonanthura ferruginea* (Less.) H.Rob. | Assapeixe branco | Medicinal | Bronchitis |
| *Gymnanthemum amygdalinum* (Delile) Sch.Bip. ex Walp. | Caferana | Medicinal | Digestion, Congestion, Liver problems |
| *Achyrocline satureioides (Lam.) DC.* | Camomila | Medicinal | Calmative |
| *Baccharis trimera* (Less.) Dr. | Carqueja | Medicinal | Stomach problems (digestion, congestion), infection |
| *Chromolaena maximilianii* (Schrad. ex DC.) R.M.King & H.Rob. | Cruzeirinho | Medicinal | Wound |
| *Tithonia diversifolia* (Hemsl.) A.Gray | Figatil/flor da amazônia | Medicinal | Liver problems, Kidney problem, Digestive |
| *Mikania glomerata* Spreng. | Guaco | Medicinal | Expectorant, Cough, Flu, Anemia |
| *Artemisia absinthium* L. | Losna | Medicinal | Digestive, Cough, Pneumonia |
| *Angeratum conyzoides* L. | Mentraste | Medicinal | cough, flu, Pneumonia, Stomach, Baby cramps and gas, Menstrual cramps |
| *Bidens pilosa* L. | Picão | Medicinal | Jaundice, Anemia |
| **Bignoniaceae** |  |  |  |
| *Jacaranda rufa* Silva Manso | Caroba | Medicinal | Anemia, cough, influenza, depurative, body injuries (sores) |
| *Handroanthus heptaphyllus* (Vell.) Mattos | Ipê roxo | Medicinal | Treatment of edema |
| *Tabebuia aurea* (Silva manso) Benth. & Hook. F. ex. S. Moore | Paratudo | Medicinal | Vermifuge, Anemia, Fortifier, cough, flu, Antibiotic |
| **Bixaceae** |  |  |  |
| *Bixa orellana* L. | Urucum, colorau | Medicinal, Food | Spice, Diabetes, Labirintitis, Cholesterol, Liver problems, Food |
| *Cochlospermum regium* (Schvank) Pelg. | Algodãozinho | Medicinal | Depurative, Skin blemishes |
| **Bromeliaceae** |  |  |  |
| *Ananas ananas* (L.) H.Karst. ex Voss | Abacaxi | Food | Food |
| *Bromelia balansae* Mez | Gravatá | Medicinal, Food | Anemia, cough, flu, food |
| **Cactaceae** |  |  |  |
| *Pereskia aculeata* Mill. | Orapronobis | Medicinal | Constipation |
| **Caricaceae** |  |  |  |
| *Carica papaya* L. | Mamão | Medicinal, Food | Laxative, Vermifuge, Cholesterol, Food |
| **Caryocaraceae** |  |  |  |
| *Caryocar brasiliense* Cambess | Pequi | Food | Food |
| **Combretaceae** |  |  |  |
| *Terminalia argentea* Mart. | Capitão | Medicinal | Flu, Infection, Diarrhea |
| *Terminalia fagifolia*Mart. | Mussambé | Medicinal | Infection, diarrhea, Cholesterol, Diabetes, Depurative, Antibiotic, Stomach problems (Gastritis, stomach ulcers, heartburn, indigestion ...) |
| **Costaceae** |  |  |  |
| *Costus spicatus* (Jacq.) Sw | Cana de macaco | Medicinal | Kidney problem, Diuretic |
| **Crassulaceae** |  |  |  |
| *Kalanchoe crenata (Andrews) Haw.* | Folha santa | Medicinal | Cholesterol, Diabetes, Cough, Flu, Expectorant |
| **Curcubitaceae** |  |  |  |
| *Cucumis anguria L.* | Maxixi | Medicinal, Food | Diabetes, Food |
| *Curcubita moschata* Dusch | Abóbora | Medicinal, Food | Food, Vermifuge |
| *Citrullus lanatus* (Thunb.) Matsum. & Nakai | Melancia | Food | Food |
| *Momordica charantia* L. | Melão de São Caetano | Medicinal | Stomach problems, internal inflammation, healing, cancer treatment, Dengue, antibiotic |
| **Dilleniaceae** |  |  |  |
| *Davilla elliptica A.St. - Hill.* | Lixeirinha | Medicinal | Edema |
| **Euphorbiaceae** |  |  |  |
| *Jatropha elliptica* (pohl) 0Ken | Batata de teiú (jalapá) | Medicinal | Digestive, Depurative, Infection of the stomach, Vitiligo, Vermífugo, Treatment of cancer, Rheumatism |
| *Synadenium grantii Hook. f.* | Jaborana | Medicinal | Cancer prevention |
| *Manihot esculenta* Crantz | Mandioca | Food | Food |
| *Croton antisyphiliticus* Mart. | Pé de perdizes | Medicinal | Infection of the uterus, Infection, Injury |
| *Croton urucurana Baill.* | Sangra d’água (cáscara sagrada) | Medicinal | Gynecological infection, Antibiotic |
| *Cnisdoscolus urens* (L) Arthur | Urtiga roxa | Medicinal | Skin conditions, Inflammation of throat, Infection, Gastritis, Depurative, anemia, Gynecological infection, Cancer treatment |
| **Fabaceae** |  |  |  |
| *Bauhinia pentandra* (Bong.) Vogel ex Stend. | Pata de vaca | Medicinal | Weight loss |
| *Amburana cearensis* (Allemão) A.C.Sm. | Amburana | Medicinal | Chronic diseases, Rheumatism |
| *Vatairea macrocarpa (Benth.) Ducke* | Angelim (Maleitoso/ Pau de bororo) | Medicinal | Depurative, Treatment of menopause, cough, flu, Constipation |
| *Anadenanthera colubrina*(Vell.) Brenan | Angico/ angico branco | Medicinal | Fever, malaria, headache. Digestion, Congestion, Diabetes, Liver problems, edema, to slim down, body aches |
| *Stryphnodendron adstringens* (Mart.) Coville | Barbatimão | Medicinal | Bronchitis, cough, respiratory problems, influenza, depurative |
| *Dipteryx alata* Vogel | Baru (cumbaru) | Medicinal | Infection, Stomach ulcer, Throat infection, Uterine infection, Vaginal astringent, Wound |
| *Copaifera langsdorffii Desf.* | Copaíba (Pau de óleo) | Medicinal, Food | Anemia, Edema, Kidney problem, Aphrodisiac, food |
| *Senna occidentalis* (L.) Link. | Fedegoso/ fedegoso roxo | Medicinal | Flu, bronchitis, cough, Anti-inflammatory |
| *Hymenaea stigonocarpa* Mart. Ex | Jatobá | Medicinal | Flu, cough, Headache, Expectorant, Body aches, fever, Pneumonia, Menopause treatment, Menstrual cramps, Postpartum uterine cleansing, Liver problems |
| *Pterodon emarginatus* Vogel | Sucupira | Medicinal, Food, Wood | Flu, Bronchitis, cough, pneumonia, anemia, Depurative, Stomach problems, Respiratory problems, Anti-inflammatory, Cancer treatment, Food, Construction |
| *Tamarindus indica* L. | Tamarindo | Medicinal | Fortifier, throat infection, sore throat, flu, depurative |
| *Leptolobium dasycarpum* Vogel | Unha danta (unha de anta) | Medicinal, Food | Laxative Cholesterol Food |
| *Anadenanthera peregrina* (L.) Speg. | Angico | Medicinal | Stomach pain, digestive, dysentery, diarrhea, Headache, Anti-inflammatory, Sinusitis, Vermifuge |
| **Krameriaceae** |  |  |  |
| *Krameria argentea* Mart. ex Spreng. | Roseta | Medicinal, Food | Spice, Cough, Sore Throat |
| **Lamiaceae** |  |  |  |
| *Rosmarinus officinalis* L. | Alecrim | Medicinal, Food | Spice, Heart problems (arrhythmia) |
| *Ocimum basilicum* L. | Alfavaca | Medicinal | Calm, fever, headache, anemia, cough, flu, bladder infection |
| *Plectranthus barbatus* Andr. | Boldo | Medicinal | Digestive |
| *Plectranthus amboinicus* (Lour.) Spreng. | Hortelã grande | Medicinal, Food | Spice, Flu, Cough, Bronchitis, Throat infection, Cancer treatment |
| *Mentha spicata* L. | Hortelã pequeno | Medicinal, Food | Spice, Influenza, Throat infection, Vermifuge |
| *Mentha pulegium* L. | Poeijo | Medicinal | Baby Cramps, Child Influenza, Flu |
| **Lauraceae** |  |  |  |
| *Persea americana* Mill. | Abacate | Medicinal, Food | Pain, kidney problem, food |
| *Cinnamomum verum J.Presl* | Canela | Medicinal | Aromatizing, cough |
| **Lecythidaceae** |  |  |  |
| *Cariniana estrellensis* (Raddi) Kuntze | Jequitibá | Medicinal | Infection in mucous membranes (stomach, mouth, cold sore), Healing mucosal lesions |
| **Loganiaceae** |  |  |  |
| *Strychnos pseudoquina* A. St.. - Hil | Quina | Medicinal | Depurative, Digestive, Anemia, Vermifuge, Cough, Headache, Appetite Stimulant, Stomach Pain, Diabetes |
| **Lythraceae** |  |  |  |
| *Lafoensia pacari* A.St.-Hil. | Mangabeira brava, didaveiro/didal | Medicinal | Stomach problems, ulcers, wounds, healing, Vermifuge, Antibiotic, Skin conditions (scabies), Prevention of prostate cancer, Treatment of breast cancer |
| **Lythraceae** |  |  |  |
| *Punica granatum* L. | Romã | Medicinal | Healing, Aphthae, Throat inflammation, Sore throat |
| **Malphighiaceae** |  |  |  |
| *Malphighia glabra L.* | Acerola | Medicinal, Food | Food, Flu, Diabetes |
| *Byrsonima* sp. | Murici roxo | Medicinal | Stomach infection, diarrhea |
| *Peixotoa cordistipula A. Juss.* | João da Costa | Medicinal | Kidney problems |
| **Malvaceae** |  |  |  |
| *Gossypium barbadense* L. | Algodão caseiro | Medicinal | Inflammation, pain, ear pain, gynecological infection, infection |
| *Waltheria americana* L. | Malva branca | Medicinal | Expectorant, Infection |
| *Guazuma ulmifolia* Lam. | Mutambá | Medicinal | Cholesterol, Uric Acid |
| *Bytteneria melastomiffolia* St. Hil | Raiz de bugre | Medicinal | Diarrhea |
| **Marantaceae** |  |  |  |
| *Ischnosiphon sp.* | Pacova | Medicinal | Calmative |
| **Meliaceae** |  |  |  |
| *Azadiractha indica* A. Juss | Nim indiano | Medicinal | Diabetes, Cholesterol, Repellent |
| **Menispermaceae** |  |  |  |
| *Cissampelos pareira* L. | Orelha de onça | Medicinal | Kidney infection, Prevention of prostate cancer |
| **Moraceae** |  |  |  |
| *Dorstenia cayapia* Vell. | Carapiá | Medicinal | Bronchitis, Vermifuge |
| *Brosimum gaudichaudii* Trécul | Mama cadela | Medicinal | Vitiligo, Infection of the uterus, Depurative, itching, Heart problems |
| *Morus nigra* L. | Amora | Medicinal, Food | Menopause, Hemorrhoid, Backache, Toothache, Food |
| **Myrtaceae** |  |  |  |
| *Psidium guajava l.* | Goiaba | Medicinal, Food | Diarrhea, Healing, Food |
| *Campomanesia adamantium* (Cambess.) O.Berg | Guavira | Food | Food |
| *Plinia cauliflora* (Mart.) O. Berg. | Jabuticaba | Food | Food |
| *Syzygium cumini* (L.) Skeels | Jamelão | Food | Food |
| *Eugenia pitanga* (O.Berg) Nied. | Pitanga | Medicinal, Food | Hypertension, Food |
| **Nyctaginaceae** |  |  |  |
| *Boerhavia diffusa* L. | Amarra pinto | Medicinal | Uric acid |
| **Passifloraceae** |  |  |  |
| *Passiflora edulis* Sims | Maracujá | Medicinal, Food | Calming, Diabetes, Food |
| **Phyllanthaceae** |  |  |  |
| *Phyllanthus niruri* L. | Quebra pedra | Medicinal | Kidney infection |
| **Phytolacaceae** |  |  |  |
| *Gallesia integrifolia* (Spreng.) Harms | Pau d'alho | Medicinal | Pain in the spine, Diabetes, Expectorant |
| **Piperaceae** |  |  |  |
| *Piper tuberculatum Jacq.* | Jaborandi | Cosmetics | Hair Treatment |
| **Poaceae** |  |  |  |
| *Zea mays subsp. mexicana* (Schrad.) Iltis | Cabelo de milho roxo | Medicinal | Urinary infection |
| *Digitaria insularis* (L.) Mez ex. Ekman | Capim amargoso | Medicinal | Kidney infection, Cicatrizante, Antibiotic, Infection of the stomach, Throat infection |
| *Cymbopogon citratus (DC.) Stapf* | Capim Cidreira | Medicinal | Calming, flu |
| *Guadua paniculata* Munro | Taboca | Medicinal | Internal healing |
| **Rubiaceae** |  |  |  |
| *Chiococca alba (L.) Hith.* | Cainca | Medicinal | Indigestion, Headache, Snakebite, Pain in the spine, backache, Constipation, Liver problems, Menopause problems |
| *Palicourea xanthophylla* M. | Douradinha | Medicinal | Diuretic, Renal problem |
| *Palicourea rigida* H.B.K. | Douradona | Medicinal | Diuretic, Renal problem |
| *Genipa americana* L. | Jenipapo | Medicinal, Food | Anemia, Diabetes, Food |
| *Cordiera edulis* (Rich.) A. Rich. Ex. DC | Marmelo de bola | Food | Food |
| *Morinda citrifolia* L. | None | Medicinal | Treatment of cancer, Foot edema |
| **Rutaceae** |  |  |  |
| *Ruta graveolens* L. | Arruda | Medicinal | Abortion, Headache, Menopause treatment, Constipation, Anticipation of menstrual flow, Postpartum uterine cleansing, Menstrual cramps, Uterine infection, Mistico |
| *Citrus sinensis* (L.) Osbeck cv. *lima* | Laranja lima | Medicinal, Food | Hypertension, Calmative, Food |
| *Citrus sinensis* (L.) Osbeck cv. *pera* | Laranjeira | Medicinal, Food | Anemia, cough, flu, headache, pneumonia, bronchitis, food |
| *Citrus x limon* (L.) Osbeck | Limão | Medicinal, Food | Appetite suppressant, Flu, Liver problems, Circulation, Memory, Food, General soap cleaning |
| *Citrus aurantifolia* (Christm.) Swingle. cv. galego | Limão galego | Medicinal | Flu |
| *Citrus bigaradia* Loisel. | Limão rosa | Medicinal, Food | Throat irritation, flu, food |
| *Citrus latifolia* Tanaka | Limão taiti | Medicinal, Food | Cough, Flu, Anemia, Food |
| *Spiranthera odoratissima* A.St.-Hil. | Manacá | Medicinal | Rheumatism, Gynecological Infection, Arthritis, Uric Acid, Depurative |
| *Citrus reticulata* Blanco | Pocã | Food | Food |
| **Sapindaceae** |  |  |  |
| *Dilodendron bipinnatum* Radlk. | Maria pobre | Medicinal | Itchy, Plant ashes for Dandruff |
| *Serjania erecta* Radk | Cinco folhas | Medicinal | Cholesterol, Backache, Constipation |
| *Magonia pubescens* St. Hil | Timbó | Medicinal | Pain in the joints |
| **Scrophulariaceae** |  |  |  |
| *Scoparia dulcis* L. | Vassourinha | Medicinal | Toothache, Body damage and pain in the body, conjunctivitis |
| **Siparunaceae** |  |  |  |
| *Siparuna guianensis* Aubl. | Negramina | Medicinal | Constipation, Sinusitis, Arthritis, Rheumatism |
| **Solanaceae** |  |  |  |
| *Solanum tuberosum* L. | Batata | Medicinal | Fever |
| *Atropa belladonna* L. | Beladona | Medicinal | Earache, Furuncle, Mumps, Erysipelas |
| *Solanum melongena* L. | Berinjela | Medicinal, Food | Appetite inhibitor, food |
| *Solanun paniculatum* L. | Jurubeba | Medicinal, Food | Liver problems, Food |
| *Capsicum* sp. | Pimenta bodinha | Medicinal, Food | Healing, spice |
| *Capsicum frutescens* L. | Pimenta malagueta | Medicinal | Erysipelas |
| **Urticaceae** |  |  |  |
| *Cecropia pachystachya* Trécul | Embaúba | Medicinal | Anti-inflammatory, Arthritis, arthrosis, Hypertension, Bronchitis, flu, cough |
| **Verbenaceae** |  |  |  |
| *Lippia alba* (Mill.) N.E.Br. ex P. Wilson | Erva cidreira | Medicinal | Calmative, Flu, Cough, Headache, Pneumonia |
| *Stachytarpheta cayennensis* (Rich.) Vahl. | Gervão | Medicinal | Flu, Expectorant, Uterine infection, Liver problems |
| **Vitaceae** |  |  |  |
| *Cissus erosa* Rich | Erva de sapo | Medicinal | Berne |
| **Vochysiaceae** |  |  |  |
| *Vochysia rufa* Mart | Pau doce | Medicinal | Diabetes, Dysentery |
| *Qualea grandiflora* Mart. | Pau terra | Medicinal | Diarrhea, Intestinal infection |
| **Zingiberaceae** |  |  |  |
| *Curcuma longa* L. | Açafrão | Medicinal | Inflammation |
| *Zingiber officinale* Roscoe | Gengibre | Medicinal | Cough, Bronchitis, Flu, Throat infection |
